# EXPERIENCES OF MUSIC LISTENING AMONG OLDER ADULTS WITH MILD COGNITIVE IMPAIRMENT

**DOI:** 10.1093/geroni/igae098.1725

**Published:** 2024-12-31

**Authors:** Claire Wang, Mengchi Li, Junxin Li

**Affiliations:** Johns Hopkins University, Baltimore, Maryland, United States; Xi’an Jiaotong University, Xi’an, Shaanxi, China (People’s Republic); Johns Hopkins School of Nursing, Baltimore, Maryland, United States

## Abstract

Emerging research highlights the cognitive benefits of music listening for older adults. The nuances of these effects, particularly concerning musical features like tempo, memorability, lyrics, and genre, remain underexplored especially among those with mild cognitive impairment (MCI). This mixed-methods study, nested within a randomized controlled crossover trial, explored these features through assessments of participants’ musical experience (Assessment of Personal Abductive Preference and Goldsmiths Musical Sophistication Index) at baseline and semi-structured interviews following a 4-week personalized music intervention (~30 self-selected songs, 1 hour/day, ≥5 days/week). Participants (n=25) were on average aged 68.0 ± 7.1 years (min: 55.0, max: 81.0), predominantly female (68%) and Black (68%). Almost half of the participants had some college or above education (44%), and about 36% of participants lived in extreme poverty. The findings highlight the significant value of music with 56.5% of participants reported routinely listening to music attentively for at least an hour each day; 42.9% described music’s role in their lives as ‘very important,’ while 47.6% rated it as ‘moderately important’; and 43.5% considered music as an indispensable part of their existence. The most popular genres reported were religious music (27.0%), followed by country/western/jazz/rock (14.8%), blues (11.1%), classical (7.4%), and folk/cultural music (3.7%). Interviews identified music essentialism, affective engagement, and feasibility as central themes, with participants preferring music that resonates with their moods or early life memories. Our findings underscore the deep-seated value of music, offering a foundation for tailoring music interventions to enhance the well-being of those with MCI.

